# Peroxisomal Localization and Circadian Regulation of Ubiquitin-Specific Protease 2

**DOI:** 10.1371/journal.pone.0047970

**Published:** 2012-11-02

**Authors:** Matthew M. Molusky, Di Ma, Katie Buelow, Lei Yin, Jiandie D. Lin

**Affiliations:** 1 Life Sciences Institute and Department of Cell & Developmental Biology, University of Michigan Medical Center, Ann Arbor, Michigan, United States of America; 2 Department of Molecular & Integrative Physiology, University of Michigan Medical Center, Ann Arbor, Michigan, United States of America; Karlsruhe Institute of Technology, Germany

## Abstract

Temporal regulation of nutrient and energy metabolism is emerging as an important aspect of metabolic homeostasis. The regulatory network that integrates the timing cues and nutritional signals to drive diurnal metabolic rhythms remains poorly defined. The 45-kDa isoform of ubiquitin-specific protease 2 (USP2-45) is a deubiquitinase that regulates hepatic gluconeogenesis and glucose metabolism. In this study, we found that USP2-45 is localized to peroxisomes in hepatocytes through a canonical peroxisome-targeting motif at its C-terminus. Clustering analysis indicates that the expression of a subset of peroxisomal genes exhibits robust diurnal rhythm in the liver. Despite this, nuclear hormone receptor PPARα, a known regulator of peroxisome gene expression, does not induce USP2-45 in hepatocytes and is dispensible for its expression during starvation. In contrast, a functional liver clock is required for the proper nutritional and circadian regulation of USP2-45 expression. At the molecular level, transcriptional coactivators PGC-1α and PGC-1β and repressor E4BP4 exert opposing effects on USP2-45 promoter activity. These studies provide insights into the subcellular localization and transcriptional regulation of a clock-controlled deubiquitinase that regulates glucose metabolism.

## Introduction

The activities of many metabolic processes in the body are precisely timed and aligned with the body clock [1], [2], [3], [4], [5]. How circadian metabolic rhythm is orchestrated and its significance in physiology and disease remain poorly understood. Recent metabolomic profiling demonstrated that numerous metabolites in circulation, including amino acids and various lipid species, exhibit robust daily cycles [6], [7], [8]. This ebb and flow of metabolites is synchronized to the oscillatory hormonal changes in circulation, most notably cortisol and leptin [9], [10]. In parallel, major metabolic pathways involved in hepatic glucose and lipid metabolism, such as glycogenolysis, gluconeogenesis, *de novo* lipogenesis, and cholesterol biosynthesis, also exhibit robust diurnal rhythms in rodents and humans [11], [12], [13], [14]. These cyclic changes of metabolic activities are accompanied with rhythmic expression of a large number of genes involved in nutrient and energy metabolism [15], [16], [17], [18], [19]. Temporal restriction of metabolic activities is emerging as an important aspect of nutrient and energy homeostasis. Perturbations of diurnal metabolic rhythms disrupt normal energy balance and also contribute to the pathogenesis of insulin resistance [20], [21], [22], [23].

In mammals, the core molecular clock is comprised of transcription activators and repressors assembled into positive and negative regulatory loops, which function to generate sustained and autonomous transcriptional rhythm [24], [25], [26]. The core components of this regulatory network receive input from light, such as in the case of the central clock residing in the suprachismatic nucleus, and diverse hormonal and nutrient cues that influence clock oscillators in peripheral tissues. We have previously demonstrated that PGC-1α is a nutritionally regulated transcriptional coactivator that regulates the expression of Bmal1, a central component of the molecular clock [27], [28]. PGC-1α is rhythmically expressed in the liver and is required for normal circadian rhythms of locomotor activity, metabolic gene expression and glucose homeostasis [28]. Disruption of PGC-1β, a close homolog of PGC-1α, also perturbs circadian regulation of locomotor activity [29]. The stability of PGC-1α is modulated by casein kinase 1δ, a core clock component, through phosphorylation and proteasome-mediated degradation [30]. In addition, the transcriptional activity of PGC-1α is further modulated by intracellular NAD^+^ levels and SIRT1-dependent deacetylation [31], suggesting that this coactivator likely serves as a regulatory hub that transduces nutrient cues to the molecular clock.

A major transcriptional target of PGC-1α that contributes to diurnal glucose regulation is ubiquitin-specific protease 2 (USP-2) [32], which belongs to a large family of deubiquitinases that regulates diverse biological processes [33], [34]. Two major isoforms of USP2 have been identified, USP2-45 and USP2-69; the former of which is highly responsive to circadian and nutritional signals. A previous study suggested that these two isoforms were generated through alternative splicing [35]. However, recent genome and transcript sequencing indicates that USP2-45 and USP2-69 are generated through the usage of distinct transcriptional start sites. USP2 has been implicated in the regulation of cell proliferation, clock function, male reproduction, and ion channel regulation [36], [37], [38], [39], [40]. While USP2 is capable of deubiquitinating a wide range of substrates, its subcellular localization has not been explored. Further, while USP2-45 expression exhibits robust circadian and nutritional regulation in an isoform-specific manner [32], [36], the molecular basis of the transcriptional regulation remains unknown. In this study, we demonstrated that both USP2 isoforms are localized to peroxisomes through a carboxyl-terminal peroxisome targeting sequence (PTS1). In addition, a balance between transcriptional coactivators and repressors dictates the transcriptional activity from the USP2-45 promoter.

## Results

### USP2 is localized to peroxisome in hepatocytes through PTS1 motif

**Figure 1:**
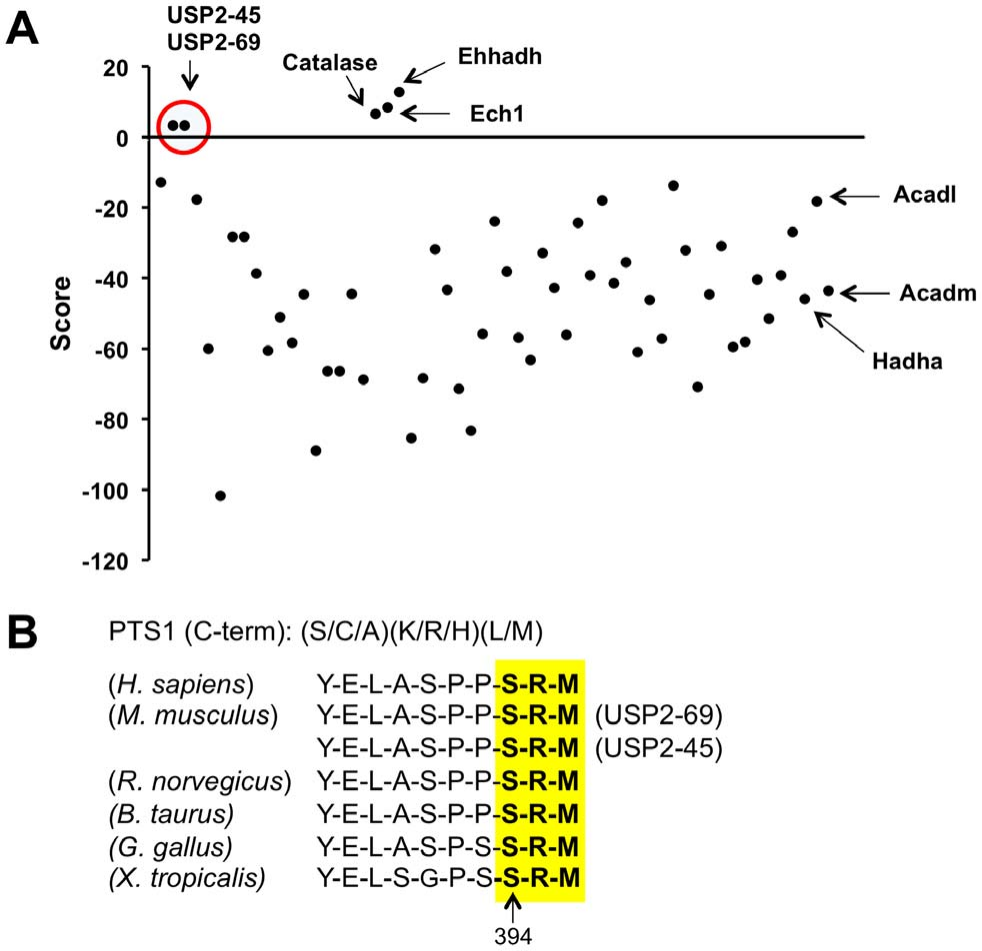
Both USP2 isoforms harbor C-terminal peroxisomal targeting sequence (PTS1). (**A**) *In Silico* prediction of PTS1 motif in the ubiquition specific protease family (USPs). Peroxisomal (Catalase, Ehhadh, Ech1) and mitochondrial (Hadha, Acadm, Acadl) proteins were included as positive and negative control, respectively. (**B**) The PTS1 motif in USP2 is conserved among human, mouse, rat, cow, chicken and frog.

We recently reported that USP2-45 regulates hepatic gluconeogenesis and circadian glucose metabolism through modulating the expression of 11β-hydroxysteroid dehydrogenase 1 in the liver [32]. However, the molecular regulation of USP2-45 with regard to its subcellular localization and the mechanisms that drive USP2-45 expression in response to nutritional and circadian cues have not been elucidated. To address these, we first employed an *in silico* approach using the prediction program WoLF PSORT (www.psort.org) to search for canonical localization signals for various subcellular compartments, including membrane, nucleus, endoplasmic reticulum, mitochondrion, peroxisome, and extracellular space [41]. Using this program, we found that both UPS2-isoforms contain a putative peroxisomal targeting sequence 1 (PTS1) in the C-terminus and are predicted to be localized to peroxisome. We next examined whether the presence of PTS1 motif is unique for USP2 among the USP family of deubiquitinating enzymes. As expected, known peroxisome-localized proteins (Catalase, Ech1, Ehhadh), but not mitochondrial proteins (Hadha, Acadm, Acadl) scored positive using the PTS1 Predictor algorithm (http://mendel.imp.ac.at/pts1/). A positive score reflects a strong probability of peroxisomal localization, while a negative score predicts a low likelihood of peroxisome localization. Remarkably, USP2-45 and USP2-69 are the only two USP family members that are predicted to contain PTS1 (**Fig. 1A**). The PTS1 motif in USP2 is highly conserved among several species, including humans, rodents, cow, chicken, and frog (**Fig. 1B**).

**Figure 2:**
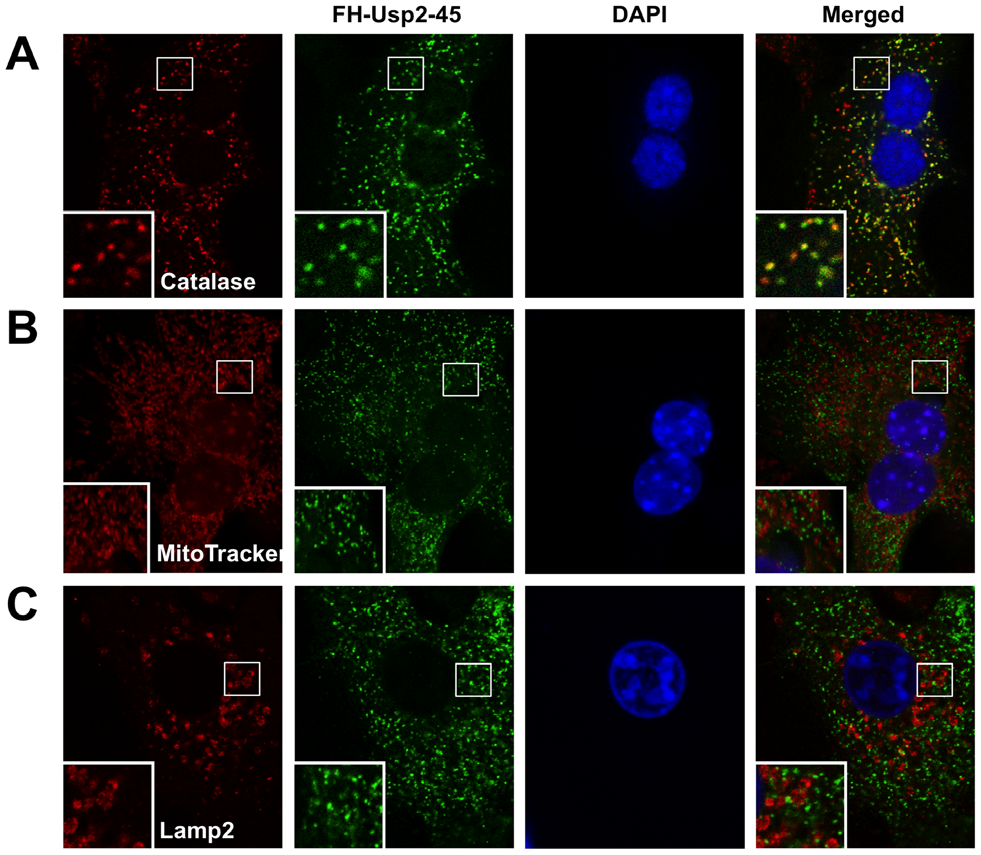
USP2-45 is localized in the peroxisome in primary hepatocytes. Primary hepatocytes were transduced with a recombinant adenovirus expressing Flag/HA-tagged USP2-45 followed by immunofluorescence staining using antibodies against Flag along with organelle markers Catalase (**A**), Mitotracker-red (**B**), or Lamp2 (**C**). Cells were counterstained with DAPI to mark the nucleus.

**Figure 3:**
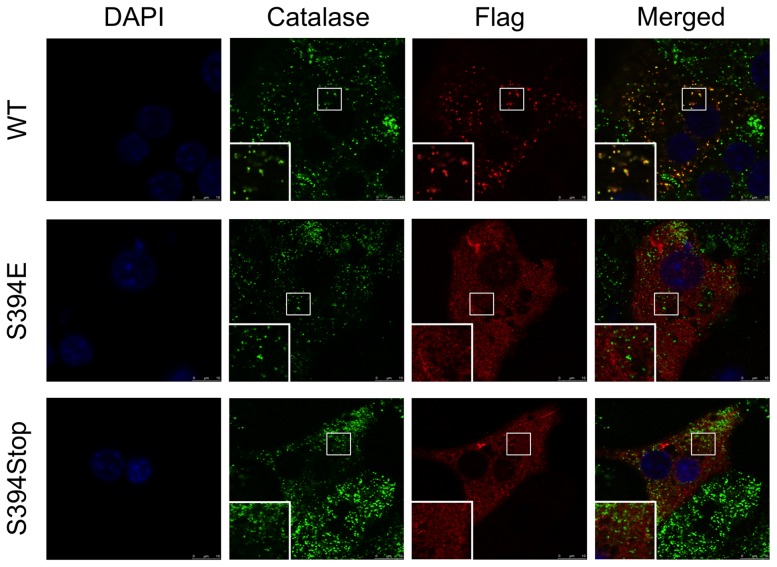
The PTS1 motif is required for peroxisomal localization of USP2-45. Immunoflurescent confocal microscopy images from primary hepatocytes transiently transfected with USP2-45 WT (*top*), USP2-45 S394E (*middle*), or USP2-45 S394Stop (*bottom*) plamids. Immunofluorescence staining was performed using Catalase and Flag antibodies. Cells were counterstained with DAPI to mark the nucleus.

To determine whether USP2-45 is localized to peroxisome, we transduced primary hepatocytes cultured on collegen-coated cover-slips with a recombinant adenovirus expressing Flag/HA-tagged USP2-45 and performed immunofluorescence staining and confocal microscopy. Co-staining of transduced hepatocytes using antibodies against Flag and catalase, a well-characterized peroxisomal marker, revealed that the punctate localization patterns of USP2-45 and catalase significantly overlapped. Most of the USP2-45-positive puncta are also positive for Catalase (**Fig. 2A**). In contrast, USP2-45 immunofluorescence does not colocalize with Mitotracker-red (**Fig. 2B**) and Lamp2 (**Fig. 2C**), which are mitochondrial and late endosomal/lysosomal markers, respectively. Interestingly, a small fraction of catalase-positive puncta exhibited relatively weak USP2-45 staining; the biological significance of this heterogeneous localization remains unknown. To determine whether the PTS1 motif on USP2-45 is required for its peroxisomal targeting, we generated a mutant construct containing a point-mutation at serine residue 394 (S394E) that disrupts peroxisomal localization in other proteins [42]. Compared to wild type USP2-45, S394E mutant no longer colocalizes with catalase (**Fig. 3**). Consistently, the peroxisomal localization of USP2-45 was also completely abrogated when a premature stop condon was introduced at serine 394 (S394Stop). Together, these observations demonstrate that USP2-45 is targeted to peroxisome in hepatocytes through its C-terminal PTS1 motif.

### Circadian regulation of peroxisomal gene expression in the liver

**Figure 4:**
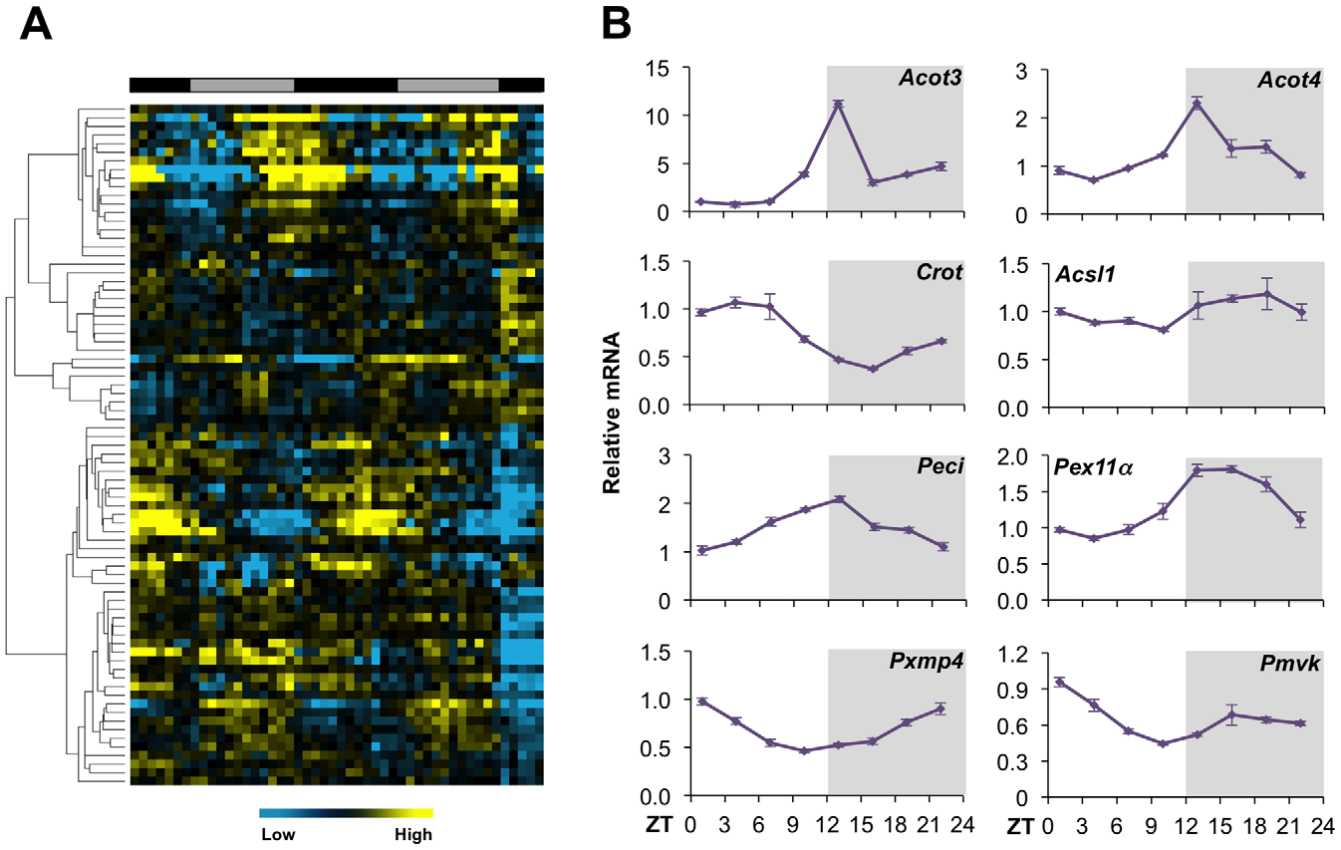
Circadian regulation of peroxisomal gene expression. (**A**) Clustering analysis of peroxisomal genes using Gene Expression Omnibus dataset GSE11923. (**B**) qPCR analysis of hepatic gene expression at different time points. Data represent mean ± stdev using total liver RNA pooled from 3–4 mice for each time point.

To determine whether circadian regulation of USP2-45 expression extends to a broader set of peroxisomal genes, we analyzed a liver microarray dataset generated in a previous transcriptional profiling study (GEO dataset GSE11923) [43]. Clustering and qPCR analyses indicated that a subset of peroxisomal genes exhibits robust diurnal rhythm in the liver (**Fig. 4A**), including those involved in peroxisomal fatty acid β-oxidation (FAO), such as acyl-CoA thioesterase 3 (Acot3), Acot4, carnitine O-octanoyltransferase (Crot), acyl-CoA synthetase long chain family member 1 (Acsl1), and peroxisome delta3, delta2-enoyl-CoA isomerase (Peci) (**Fig. 4B**). Similarly, several genes encoding the structural proteins of peroxisome also exhibit rhythmic mRNA expression, including peroxisome biogenesis factor 11α (Pex11α) and peroxisome membrane protein 4 (Pxmp4). The expression of phosphomevalonate kinase (Pmvk), a peroxisome-localized protein in cholesterol biosynthesis pathway, is also diurnally regulated. These observations are consistent with previous morphometric studies in hepatocytes [44], and raise an intriguing possibility that key aspects of peroxisomal function are under the regulation of biological clock.

**Figure 5:**
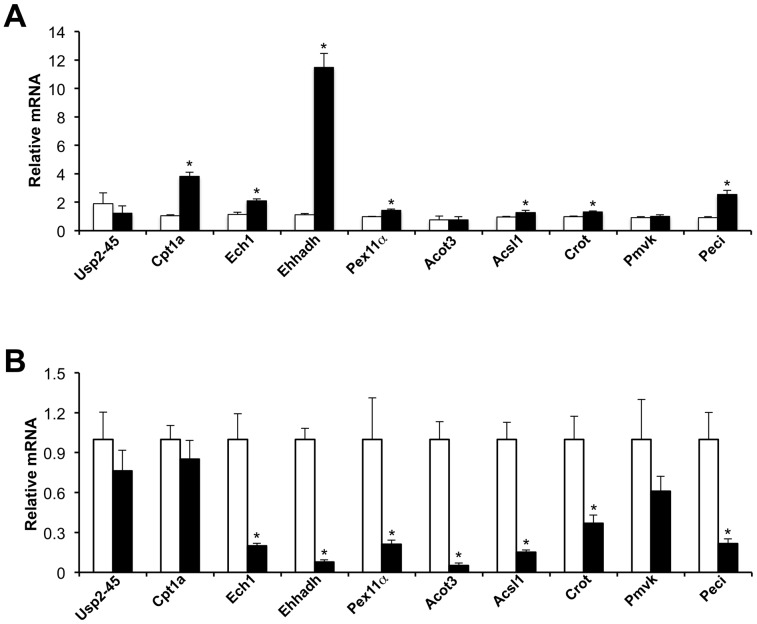
PPARα has modest effects on USP2-45 gene expression. (**A**) Gene expression analysis of primary hepatocytes treated with vehicle (open bars) or GW7647 (filled bars) for 18 hrs. Data were normalized to vehicle-treated samples and represent mean ± stdev of one representative treatment performed in triplicate. * p<0.05, GW7647 vs. vehicle. (**B**) Fasting liver gene expression in WT (open bars, n = 4) or PPARα null (filled bars, n = 6) mice. Data represent mean ± sem. * p<0.05, null vs. WT.

In mammals, peroxisomes are responsible for the bulk oxidation of branched-chain and very long-chain fatty acids. Peroxisomal fat oxidation is significantly induced in the liver during starvation through transcriptional activation of genes involved in fatty acid β-oxidation by the nuclear receptor PPARα [45], [46]. Synthetic agonists for PPARα stimulate the expression of genes involved in peroxisomal and mitochondrial fat oxidation, whereas deficiency of PPARα signaling impairs the induction of FAO genes and results in hepatic steatosis following starvation [47], [48]. The expression of PPARα itself is diurnally regulated in the liver [49]. We previously reported that USP2-45 is highly induced in liver during starvation [32]. To detemine whether PPARα signaling plays a role in nutritional regulation of USP2-45, we treated cultured primary hepatocytes with GW7647, a potent agonist specific for PPARα. Gene expression analyses indicated that mRNA expression of several known PPARα target genes was readily induced by GW7647 including Ehhadh, Ech1, Acsl1, Crot, Peci, Pex11α, and carnitine palmitoyltransferase 1 (Cpt1a), a rate-limiting enzyme for mitochondrial fat oxidation (**Fig. 5A**). In contrast, USP2-45 mRNA levels remained largely unchanged following GW7647 treatments. We observed modest induction of Acot3 and Pmvk in response to PPARα activation. To further determine whether PPARα is required for USP2-45 expression in fasted liver, we examined hepatic gene expression in wild type and PPARα null mice after overnight starvation. As expected, mRNA expression of several FAO genes, including Ech1, Ehhadh, Acot3, Acsl1, Crot, and Peci, was significantly lower in PPARα null mouse livers compared to control (**Fig. 5B**). In contrast, USP2-45 mRNA levels remained similar between these two groups. Further, PPARα deficiency has modest effects on hepatic USP2-45 expression under fed condition (data not shown). These gain- and loss-of-function studies strongly suggest PPARα is not required for the nutritional regulation of USP2-45 in the liver.

### A functional liver clock is required for nutritional and circadian regulation of USP2-45 expression

**Figure 6:**
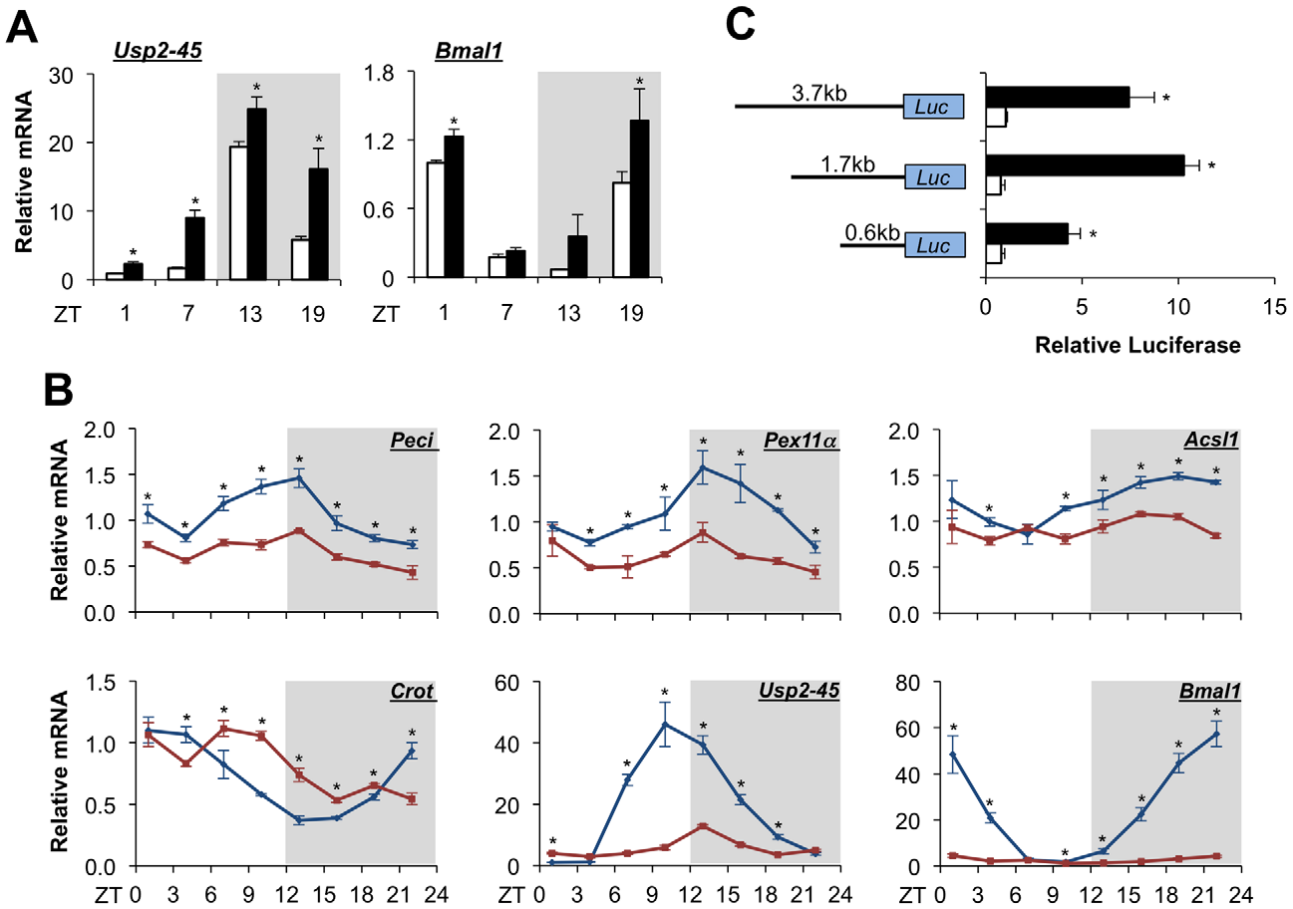
Bmal1 is required for circadian regulation of USP2-45 in the liver. (**A**) USP2-45 mRNA expression in the livers from fed (open) and fasted (filled) mice of 3–4 months of age harvested at indicated time points. Data represent mean ± stdev using pooled liver RNA (n = 3–4). *p<0.05 fed vs. fasted. (**B**) Circadian regulation of hepatic gene expression in control flox/flox (blue) and Bmal1 liver-specific null mice (red). Data represent mean ± stdev using pooled liver RNA (n = 3–5). *p<0.05 flox/flox vs. LKO. (**C**) Reporter gene assay using USP2-45 promoter constructs with vector (open) or Bmal1 and Clock (filled). Shown is a representative experiment performed in triplicates. *p<0.05 vector vs. Bmal1 and Clock.

We recently demonstrated that hepatic USP2-45 expression is induced by starvation and responds to circadian signals. It remains unknown whether circadian regulation of USP2-45 is mediated in part by nutritional cues. To assess the relative contribution of clock and nutritional signals in driving USP2-45 expression, we analyzed hepatic gene expression in wild type mice at four different time points following 24-hr starvation. As expected, USP2-45 mRNA levels are elevated at all time points in the livers from fasted mice compared to respective fed control (**Fig. 6A**). Rhythmic expression of USP2-45 persists under both fed and fasted conditions, suggesting that nutritional and circadian timing cues are likely distinct and converge to regulate USP2-45 expression.

To determine whether the molecular clock regulates diurnal expression of peroxisomal genes, we analyzed hepatic gene expression in control (flox/flox) and liver-specific Bmal1 null (Bmal1 LKO) mice sacrificed every 3 hrs through a light/dark cycle. As shown in Fig. 6B, Bmal1 deficiency significantly perturbs the amplitude and/or phase of the circadian rhythm of peroxisomal genes, such as Peci, Pex11α, Acsl1, and Crot. Rhythmic expression of USP2-45 is also severely dampened in mouse livers lacking a function clock. Recent global chromatin occupancy studies revealed that Bmal1 binds to putative regulatory elements on many hepatic genes [50], [51]. In fact, two such sites were found on the proximal promoter of USP2-45 (chr9:43891798–43891848) and intron 1 (chr9:43894103–43894153). To test whether Bmal1 regulates USP2-45 expression, we constructed luciferase reporters containing varying lengths of the proximal USP2-45 promoter and performed reporter gene studies in transiently transfected cells. The putative E-box found in the proximal USP2-45 promoter is present in the 1.7 kb and 3.7 kb constructs. Cotransfection of Bmal1 and Clock augments luciferase activity in these reporter constructs, and to a lesser extent, in 0.6 kb reporter (**Fig. 6C**). As a positive control Per1-luciferase repoter was significantly induced by Bmal1 and Clock heterodimer (data not show). This data suggests that the Bmal1/Clock transcritional complex regulates USP2-45 promoter activity.

**Figure 7:**
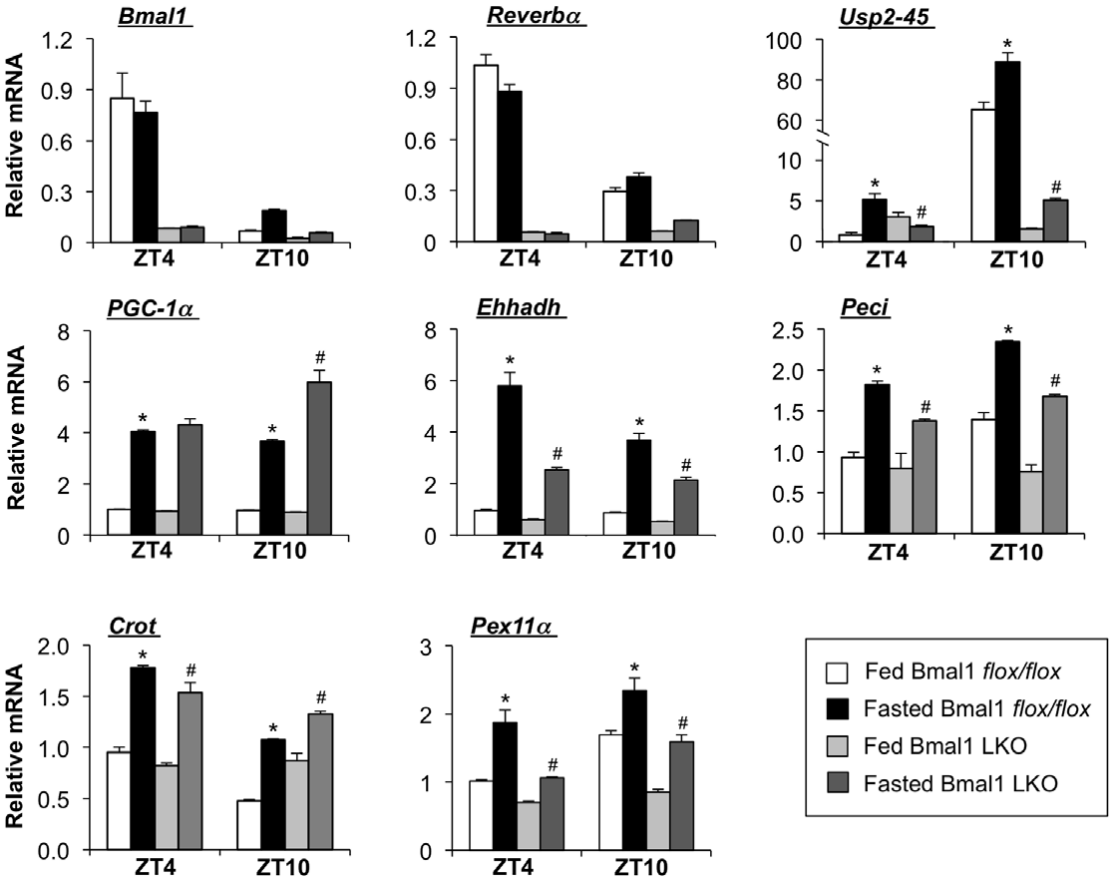
Bmal1 is required for nutritional regulation of peroxisomal genes in the liver. qPCR analysis of hepatic gene expression in Bmal1 flox/flox (fed, n = 4; fasted, n = 5) and Bmal1 LKO (fed, n = 4; fasted, n = 4) mice harvested at ZT4 and ZT10, as indicated. Data represent mean ± sem. * p<0.05, fed vs. fasted; # p<0.05, fasted flox/flox vs. fasted LKO.

To determine whether the liver clock is also required for the induction of USP2-45 expression in the starvation state, we subjected control and Bmal1 LKO mice to food deprivation for 24 hrs and examined hepatic gene expression. As expected, the expression of clock genes, including Rev-erbα was dysregulated in Bmal1-deficient livers under both fed and fasted conditions at ZT4 and ZT10 (**Fig. 7**). USP2-45 mRNA expression is robustly induced in the control liver. However, this induction was severely blunted in Bmal1 LKO livers due to its elevated expression at this time point (ZT4) in the fed state accompanied with a lack of induction in response to starvation. The induction of USP2-45 expression in response to starvation is also impaired in Bmal1 LKO animals at ZT10, when USP2-45 is near or at its zenith. The induction of several peroxisomal and mitochondrial FAO genes, including Ehhadh, Peci, Crot, and Pex11α, was also significantly impaired in the liver lacking a functional clock. These results strongly suggest that liver clock is required for circadian and nutritional regulation of peroxisomal genes in a tissue-autonomous manner.

### Antagonistic role of the PGC-1 coactivators and E4BP4 in the regulation of USP2-45 transcription

**Figure 8:**
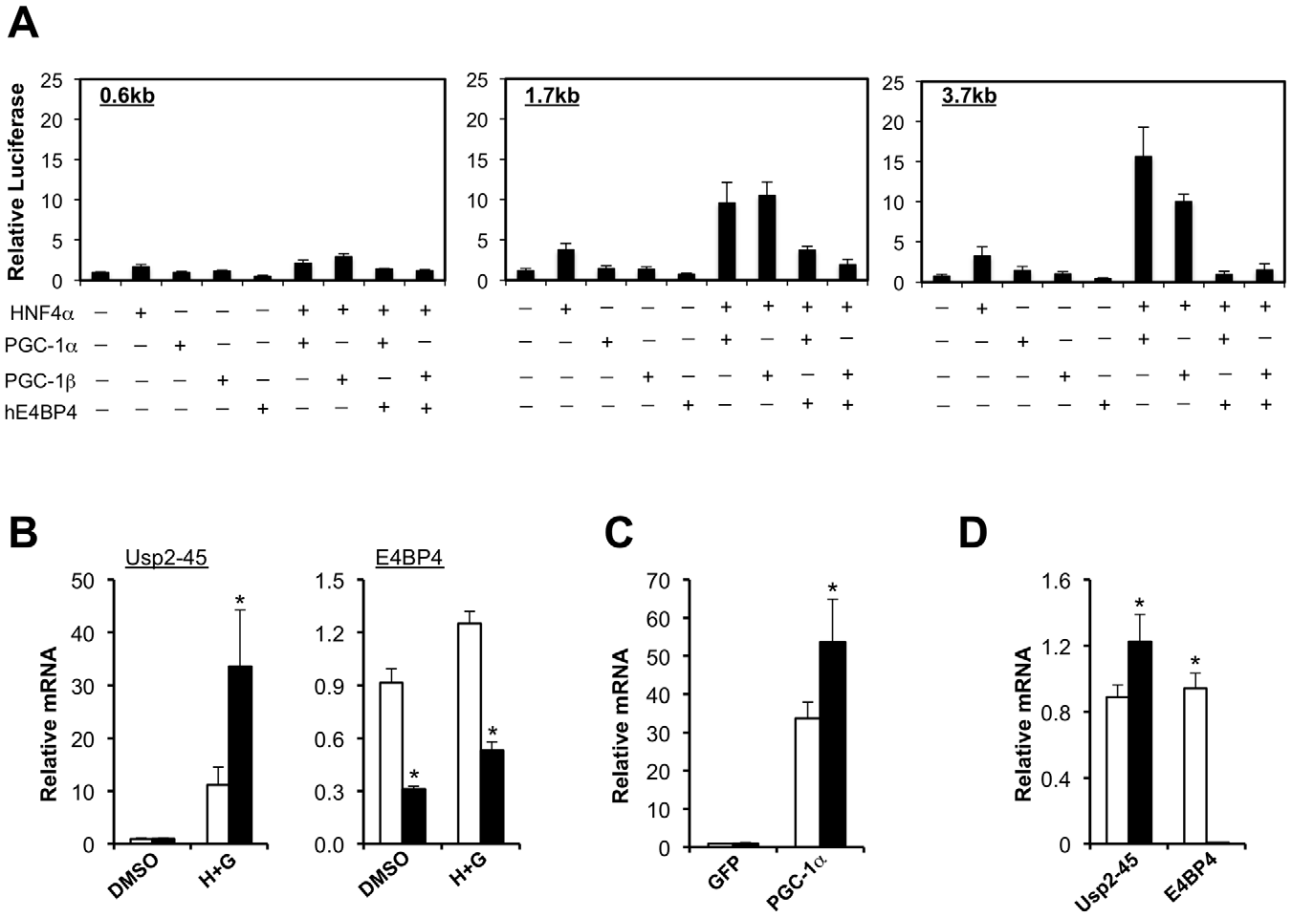
PGC-1 coactivators and E4BP4 have opposing effects on USP2-45 gene transcription. (**A**) Luciferase gene assays using the 0.6 kb (*left*), 1.7 kb (*middle*), or 3.7 kb (*right*) constructs transiently transfected with indicated plasmids. Data represent mean ± stdev of one representive experiment performed in triplicate wells. (**B**) qPCR analysis of primary hepatocytes transduced with recombinant adenoviruses expressing control shRNA (open bars) or E4BP4 shRNA (filled bars) and treated with vehicle (DMSO) or hydrocortisone (1 µM) plus glucagon (20 nM) (H+G). (**C**) qPCR analysis of primary hepatocytes transduced with Ad-PGC-1α adenovirus in the presence of adenoviruses expressing control shRNA (open bars) or E4BP4 shRNA (filled bars). Data in B–C represent mean ± stdev of one experiment performed in qualdriplates. * p<0.05, control vs. E4BP4 shRNA. (**D**) Liver USP2-45 expression in WT (open bars, n = 5) and E4BP4 null (filled bars, n = 5) mice harvested at ZT4. Data represent mean ± sem. * p<0.05 WT vs. null.

The PGC-1 family of transcriptional coactivators regulates several core metabolic pathways, including mitochondrial biogenesis, fatty acid β-oxidation, gluconeogenesis, and lipoprotein metabolism [52], [53]. By regulating the expression of core clock genes, PGC-1α also integrates circadian and nutritional cues and coordinates diurnal nutrient and energy metabolism [28]. We recently reported that PGC-1α and PGC-1β robustly activates the expression of USP2-45 in the liver and in cultured hepatocytes in an isoform-specific manner [32]. PGC-1 coactivators physically interact with selective transcription factors and stimulate the transcription of relevant target genes. In the context of hepatic gene regulation, nuclear hormone receptor HNF4α has been demonstrated to play a particularly important role [54], [55]. To determine whether HNF4α could mediate the induction of USP2-45 by PGC-1, we performed reporter gene studies in transiently transfected cells. We found that HNF4α synergized with both PGC-1α and PGC-1β when cotransfected with reporter genes containing 1.7 kb and 3.7 kb, but not 0.6 kb, fragment of the proximal USP2-45 promoter (**Fig. 8A**). Cotransfection of E4BP4, a circadian-regulated transcriptional repressor, significantly reduces the induction of USP2-45 reporter gene expression by the combination of HNF4α and PGC-1α or PGC-1β. In contrast, Rev-erbα and Rev-erbβ have modest effects on USP2-45 promoter activity in these studies (data not shown).

Consistent with reporter gene studies, we found that hormonal induction of endogenous USP2-45 gene expression in hepatocytes was significantly enhanced when E4BP4 is knocked down (**Fig. 8B**). Similarly, the stimulation of USP2-45 expression by PGC-1α was also enhanced when the hepatocytes were transduced with a recombinant adenovirus expressing shRNA targeting E4BP4 (**Fig. 8C**). Further, E4BP4 null livers showed a significant increase in USP2-45 mRNA expression (**Fig. 8D**). Taken together, these results illustrate that PGC-1 coactivators and E4BP4 play an antagonistic role in the transcriptional regulation of USP2-45.

## Discussion

To summarize, we show here that USP2 is localized to peroxisomes in the cell through a conserved PTS1 motif. The presence of a PTS1 motif is unique for USP2 among the members of the large family of deubiquitinating enzymes. Similar to USP2-45, the expression of a subset of peroxisomal genes exhibits robust diurnal rhythm. While PPARα signaling is not required for the induction of USP2-45 expression in the liver in response to starvation, an intact liver clock appears to be indispensible for its proper nutritional and circadian regulation. At the molecular level, PGC-1 coactivators robustly stimulate USP2-45 promoter activity through coactivating HNF4α. Further, E4BP4 is a clock-regulated transcriptional repressor that plays a dominant role in negatively regulating USP2-45 gene expression.

An unexpected finding is that USP-2 is localized primarily to the peroxisomal compartment. Peroxisome is an organelle best known for its role in β-oxidation of fatty acids, particularly very long-chain and branched chain fatty acids. The prominent role of peroxisomal fatty acid oxidation is supported by the identification of inborn human peroxisomal disorders that are attributed to mutations of genes involved in peroxisomal biogenesis [56]. In addition, mice deficient in PPARα failed to activate peroxisomal gene expression following prolonged starvation and developed severe hepatic steatosis accompanied by lower ketones levels in circulation [47], [48]. Peroxisome biogenesis and maintenance depend on the import of numerous membrane and matrix proteins that are encoded by the nuclear genome [57], [58]. The exact molecular machineries that mediate the transport of folded proteins and protein complexes into peroxisome remain largely unknown. However, previous studies have demonstrated that ubiquitination of Pex5, a cellular receptor that recognizes PTS1 motif, plays an important role in peroxisomal protein import [59], [60], [61]. Pex5 can undergo polyubiquitination and monoubiquitination that are mediated by distinct ubiquitin-ligase complexes. Reversible monoubiquitination of Pex5 plays a critical role in driving peroxisomal protein import cycles [57], [62]. However, the identity of deubiquitinase that is responsible for removing the monoubiquitin chain from Pex5 remains elusive. The prominent localization of USP2 to peroxisome suggests that this factor may be a putative Pex5 deubiquitinase. It is possible that USP2 itself is shuttled between the peroxisomal compartment and other cellular locations. In fact, nuclear transcription factors and membrane proteins are known to serve as substrates for USP2-mediated deubiquitination [36], [37], [38], [39], [40].

PPARα is a nuclear hormone receptor that regulates the expression of many peroxisomal genes, particularly those involved in fatty acid β-oxidation. The transcriptional activity of PPARα is increased in the liver during fasting, in part due to the recruitment of cofactors such as PGC-1α, BAF60a, Lipin 1, SIRT1, and TBL1 [63], [64], [65], [66]. Interestingly, mice deficient in PPARα have normal induction of USP2-45 expression in response to starvation, suggesting that redundant pathways are able to compensate for PPARα deficiency. In contrast, liver-specific deficiency of Bmal1 results in profound dysregulation of USP2-45 and other metabolic genes, including those involved in peroxisomal fatty acid β-oxidation. Survey of transcriptional partners for PGC-1α and PGC-1β revealed HNF4α as a potential factor in the regulation of USP2-45 gene transcription. E4BP4, but not other repressors including Rev-erbα and Rev-erbβ, strongly represses USP2-45 promoter activity. As the expression of E4BP4 itself is diurnally regulated [67], it is possible that cyclic E4BP4 activity may antagonize positive regulatory signals that together drive rhythmic expression of USP2-45.

## Materials and Methods

### Cultured primary hepatocytes

Primary hepatocytes were isolated from C57/Bl6J mice using collagenase digestion and maintained in Dulbecco Modified Eagle Medium (DMEM) supplemented with 10% bovine growth serum and antibiotics at 37°C and 5% CO_2_. Cells were switched to DMEM supplemented with 0.1% BSA for 16–24 hrs before treatments with hydrocortisone (1 µM) and glucagon (20 nM) for 3 hrs. Recombinant adenoviruses were generated using AdEasy adenoviral vector (Stratagene). Hepatocytes were transduced for 48 hrs at similar moiety of infection before RNA isolation and gene expression analysis. For qPCR analysis, total RNA was isolated from transduced hepatocytes or liver tissues using Trizol, reversed transcribed, and analyzed by quantitative PCR using the SYBR Green method.

### Immunofluorescence confocal microscopy

Primary hepatocytes were seeded on collagen coated cover-slips, transduced with Flag/HA-tagged USP2-45 adenovirus, fixed, and stained with anti-Flag and anti-catalase, Mitotracker-red™ (Invitrogen), or anti-Lamp2 followed by incubation with Alexafluor secondary antibodies. Slides were counter-stained with DAPI and analyzed by confocal microscopy. Hepatocyte transfection was performed using polyethyleneimine (PEI, Polysciences, Inc).

### Luciferase Assay

USP2-45 promoter constructs were cloned by PCR and subcloned into pGL3-Basic luciferase vector (Promega). For transient transfection, BOSC cells were were transiently transfected with reporter constructs (10–20 ng per well) in the presence of various plasmids. Cells were harvested 48 hours post-transfection using the BD Moonlight™ luciferase assay system for quantitation of luciferase activity using a Molecular Devices LMax luminometer.

### *In Vivo* Mouse Experiments

Wild-type C57/BL6J mice were either obtained from Jackson laboratories or through an in-house wild-type breeding colony and were kept on a 12∶12 light-dark (LD) cycle with food and water freely available. Starting at 7am (ZT1) 3–5 mice were sacrificed, using carbon dioxide gas chamber, every 4 hrs for a 24 hr period of time. Tissues were collected and frozen in liquid nitrogen. Samples were stored at −80°C until processed for RNA. Bmal1 liver specific knockout mice were treated with a similar protocol for circadian studies, using flox/flox mice as controls. For feeding studies all mice strains were on a 12∶12 LD cycle with water freely available. Bmal1 liver-specific knockout mice (Bmal1 LKO), along with flox/flox control mice, were fasted over-night (∼16 hrs) and harvested at 10am (ZT4) and 4pm (ZT10). For fasting circadian studies, mice were fasted for 24 hrs and harvested at ZT 1, 7, 13, and 19. For PPARα knockout animals, mice were fasted for 24 hrs and sacrificed at 4pm. All procedures for animal studies were approved by the University Committee on Use and Care of Animals (UCUCA) at the University of Michigan.

## References

[1] Wijnen H, Young MW (2006) Interplay of circadian clocks and metabolic rhythms. Annual review of genetics 40: 409–448. 10.1146/annurev.genet.40.110405.090603 17094740

[2] Bellet MM, Sassone-Corsi P (2010) Mammalian circadian clock and metabolism - the epigenetic link. Journal of cell science 123: 3837–3848. 21048160 10.1242/jcs.051649 PMC2972271

[3] Asher G, Schibler U (2011) Crosstalk between components of circadian and metabolic cycles in mammals. Cell Metab 13: 125–137. 21284980 10.1016/j.cmet.2011.01.006

[4] Green CB, Takahashi JS, Bass J (2008) The meter of metabolism. Cell 134: 728–742. 18775307 10.1016/j.cell.2008.08.022 PMC3760165

[5] Rutter J, Reick M, McKnight SL (2002) Metabolism and the control of circadian rhythms. Annu Rev Biochem 71: 307–331. 12045099 10.1146/annurev.biochem.71.090501.142857

[6] Minami Y, Kasukawa T, Kakazu Y, Iigo M, Sugimoto M, et al (2009) Measurement of internal body time by blood metabolomics. Proc Natl Acad Sci U S A 106: 9890–9895. 19487679 10.1073/pnas.0900617106 PMC2689311

[7] Dallmann R, Viola AU, Tarokh L, Cajochen C, Brown SA (2012) The human circadian metabolome. Proceedings of the National Academy of Sciences of the United States of America 109: 2625–2629. 22308371 10.1073/pnas.1114410109 PMC3289302

[8] Eckel-Mahan KL, Patel VR, Mohney RP, Vignola KS, Baldi P, et al (2012) Coordination of the transcriptome and metabolome by the circadian clock. Proceedings of the National Academy of Sciences of the United States of America 10.1073/pnas.1118726109 PMC3325727 22431615

[9] Orth DN, Island DP (1969) Light synchronization of the circadian rhythm in plasma cortisol (17-OHCS) concentration in man. The Journal of clinical endocrinology and metabolism 29: 479–486. 5779245 10.1210/jcem-29-4-479

[10] Schoeller DA, Cella LK, Sinha MK, Caro JF (1997) Entrainment of the diurnal rhythm of plasma leptin to meal timing. J Clin Invest 100: 1882–1887. 9312190 10.1172/JCI119717 PMC508375

[11] Phillips LJ, Berry LJ (1970) Circadian rhythm of mouse liver phosphoenolpyruvate carboxykinase. The American journal of physiology 218: 1440–1444. 4314570 10.1152/ajplegacy.1970.218.5.1440

[12] Sollberger A (1964) The Control of Circadian Glycogen Rhythms. Annals of the New York Academy of Sciences 117: 519–554. 14196661 10.1111/j.1749-6632.1964.tb48204.x

[13] Cella LK, Van Cauter E, Schoeller DA (1995) Diurnal rhythmicity of human cholesterol synthesis: normal pattern and adaptation to simulated “jet lag”. Am J Physiol 269: E489–498. 7573426 10.1152/ajpendo.1995.269.3.E489

[14] Munday MR, Williamson DH (1983) Diurnal variations in food intake and in lipogenesis in mammary gland and liver of lactating rats. The Biochemical journal 214: 183–187. 6137213 10.1042/bj2140183 PMC1152224

[15] McCarthy JJ, Andrews JL, McDearmon EL, Campbell KS, Barber BK, et al (2007) Identification of the circadian transcriptome in adult mouse skeletal muscle. Physiological genomics 31: 86–95. 17550994 10.1152/physiolgenomics.00066.2007 PMC6080860

[16] Panda S, Antoch MP, Miller BH, Su AI, Schook AB, et al (2002) Coordinated transcription of key pathways in the mouse by the circadian clock. Cell 109: 307–320. 12015981 10.1016/s0092-8674(02)00722-5

[17] Storch KF, Lipan O, Leykin I, Viswanathan N, Davis FC, et al (2002) Extensive and divergent circadian gene expression in liver and heart. Nature 417: 78–83. 11967526 10.1038/nature744

[18] Ueda HR, Chen W, Adachi A, Wakamatsu H, Hayashi S, et al (2002) A transcription factor response element for gene expression during circadian night. Nature 418: 534–539. 12152080 10.1038/nature00906

[19] Zvonic S, Ptitsyn AA, Conrad SA, Scott LK, Floyd ZE, et al (2006) Characterization of peripheral circadian clocks in adipose tissues. Diabetes 55: 962–970. 16567517 10.2337/diabetes.55.04.06.db05-0873

[20] Leproult R, Van Cauter E (2010) Role of sleep and sleep loss in hormonal release and metabolism. Endocr Dev 17: 11–21. 19955752 10.1159/000262524 PMC3065172

[21] Scheer FA, Hilton MF, Mantzoros CS, Shea SA (2009) Adverse metabolic and cardiovascular consequences of circadian misalignment. Proc Natl Acad Sci U S A 106: 4453–4458. 19255424 10.1073/pnas.0808180106 PMC2657421

[22] Spiegel K, Leproult R, Van Cauter E (1999) Impact of sleep debt on metabolic and endocrine function. Lancet 354: 1435–1439. 10543671 10.1016/S0140-6736(99)01376-8

[23] Turek FW, Joshu C, Kohsaka A, Lin E, Ivanova G, et al (2005) Obesity and metabolic syndrome in circadian Clock mutant mice. Science 308: 1043–1045. 15845877 10.1126/science.1108750 PMC3764501

[24] Ukai H, Ueda HR (2010) Systems biology of mammalian circadian clocks. Annual review of physiology 72: 579–603. 10.1146/annurev-physiol-073109-130051 20148689

[25] Dibner C, Schibler U, Albrecht U (2010) The mammalian circadian timing system: organization and coordination of central and peripheral clocks. Annual review of physiology 72: 517–549. 10.1146/annurev-physiol-021909-135821 20148687

[26] Welsh DK, Takahashi JS, Kay SA (2010) Suprachiasmatic nucleus: cell autonomy and network properties. Annual review of physiology 72: 551–577. 10.1146/annurev-physiol-021909-135919 PMC3758475 20148688

[27] Li S, Lin JD (2009) Molecular control of circadian metabolic rhythms. Journal of applied physiology 107: 1959–1964. 19574505 10.1152/japplphysiol.00467.2009

[28] Liu C, Li S, Liu T, Borjigin J, Lin JD (2007) Transcriptional coactivator PGC-1alpha integrates the mammalian clock and energy metabolism. Nature 447: 477–481. 17476214 10.1038/nature05767

[29] Sonoda J, Mehl IR, Chong LW, Nofsinger RR, Evans RM (2007) PGC-1beta controls mitochondrial metabolism to modulate circadian activity, adaptive thermogenesis, and hepatic steatosis. Proceedings of the National Academy of Sciences of the United States of America 104: 5223–5228. 17360356 10.1073/pnas.0611623104 PMC1829290

[30] Li S, Chen XW, Yu L, Saltiel AR, Lin JD (2011) Circadian Metabolic Regulation through Crosstalk between Casein Kinase 1delta and Transcriptional Coactivator PGC-1alpha. Mol Endocrinol 25: 2084–2093. 22052997 10.1210/me.2011-1227 PMC3231836

[31] Rodgers JT, Lerin C, Haas W, Gygi SP, Spiegelman BM, et al (2005) Nutrient control of glucose homeostasis through a complex of PGC-1alpha and SIRT1. Nature 434: 113–118. 15744310 10.1038/nature03354

[32] Molusky MM, Li S, Ma D, Yu L, Lin JD (2012) Ubiquitin-Specific Protease 2 Regulates Hepatic Gluconeogenesis and Diurnal Glucose Metabolism Through 11beta-Hydroxysteroid Dehydrogenase 1. Diabetes 10.2337/db11-0970 PMC3331773 22447855

[33] Komander D, Clague MJ, Urbe S (2009) Breaking the chains: structure and function of the deubiquitinases. Nature reviews Molecular cell biology 10: 550–563. 19626045 10.1038/nrm2731

[34] Reyes-Turcu FE, Ventii KH, Wilkinson KD (2009) Regulation and cellular roles of ubiquitin-specific deubiquitinating enzymes. Annual review of biochemistry 78: 363–397. 10.1146/annurev.biochem.78.082307.091526 PMC2734102 19489724

[35] Park KC, Kim JH, Choi EJ, Min SW, Rhee S, et al (2002) Antagonistic regulation of myogenesis by two deubiquitinating enzymes, UBP45 and UBP69. Proceedings of the National Academy of Sciences of the United States of America 99: 9733–9738. 12107281 10.1073/pnas.152011799 PMC124996

[36] Scoma HD, Humby M, Yadav G, Zhang Q, Fogerty J, et al (2011) The de-ubiquitinylating enzyme, USP2, is associated with the circadian clockwork and regulates its sensitivity to light. PloS one 6: e25382. 21966515 10.1371/journal.pone.0025382 PMC3179520

[37] Bedard N, Yang Y, Gregory M, Cyr DG, Suzuki J, et al (2011) Mice lacking the USP2 deubiquitinating enzyme have severe male subfertility associated with defects in fertilization and sperm motility. Biology of reproduction 85: 594–604. 21543767 10.1095/biolreprod.110.088542 PMC4480438

[38] Shan J, Zhao W, Gu W (2009) Suppression of cancer cell growth by promoting cyclin D1 degradation. Molecular cell 36: 469–476. 19917254 10.1016/j.molcel.2009.10.018 PMC2856324

[39] Stevenson LF, Sparks A, Allende-Vega N, Xirodimas DP, Lane DP, et al (2007) The deubiquitinating enzyme USP2a regulates the p53 pathway by targeting Mdm2. The EMBO journal 26: 976–986. 17290220 10.1038/sj.emboj.7601567 PMC1852834

[40] Fakitsas P, Adam G, Daidie D, van Bemmelen MX, Fouladkou F, et al (2007) Early aldosterone-induced gene product regulates the epithelial sodium channel by deubiquitylation. Journal of the American Society of Nephrology : JASN 18: 1084–1092. 17344426 10.1681/ASN.2006080902

[41] Horton P, Park KJ, Obayashi T, Fujita N, Harada H, et al (2007) WoLF PSORT: protein localization predictor. Nucleic acids research 35: W585–587. 17517783 10.1093/nar/gkm259 PMC1933216

[42] Gould SJ, Keller GA, Hosken N, Wilkinson J, Subramani S (1989) A conserved tripeptide sorts proteins to peroxisomes. The Journal of cell biology 108: 1657–1664. 2654139 10.1083/jcb.108.5.1657 PMC2115556

[43] Hughes ME, DiTacchio L, Hayes KR, Vollmers C, Pulivarthy S, et al (2009) Harmonics of circadian gene transcription in mammals. PLoS genetics 5: e1000442. 19343201 10.1371/journal.pgen.1000442 PMC2654964

[44] Uchiyama Y, Asari A (1984) A morphometric study of the variations in subcellular structures of rat hepatocytes during 24 hours. Cell and tissue research 236: 305–315. 6733756 10.1007/BF00214231

[45] Mandard S, Muller M, Kersten S (2004) Peroxisome proliferator-activated receptor alpha target genes. Cellular and molecular life sciences : CMLS 61: 393–416. 14999402 10.1007/s00018-003-3216-3 PMC11138883

[46] Lefebvre P, Chinetti G, Fruchart JC, Staels B (2006) Sorting out the roles of PPAR alpha in energy metabolism and vascular homeostasis. The Journal of clinical investigation 116: 571–580. 16511589 10.1172/JCI27989 PMC1386122

[47] Hashimoto T, Cook WS, Qi C, Yeldandi AV, Reddy JK, et al (2000) Defect in peroxisome proliferator-activated receptor alpha-inducible fatty acid oxidation determines the severity of hepatic steatosis in response to fasting. The Journal of biological chemistry 275: 28918–28928. 10844002 10.1074/jbc.M910350199

[48] Kersten S, Seydoux J, Peters JM, Gonzalez FJ, Desvergne B, et al (1999) Peroxisome proliferator-activated receptor alpha mediates the adaptive response to fasting. The Journal of clinical investigation 103: 1489–1498. 10359558 10.1172/JCI6223 PMC408372

[49] Lemberger T, Saladin R, Vazquez M, Assimacopoulos F, Staels B, et al (1996) Expression of the peroxisome proliferator-activated receptor alpha gene is stimulated by stress and follows a diurnal rhythm. The Journal of biological chemistry 271: 1764–1769. 8576180 10.1074/jbc.271.3.1764

[50] Hatanaka F, Matsubara C, Myung J, Yoritaka T, Kamimura N, et al (2010) Genome-wide profiling of the core clock protein BMAL1 targets reveals a strict relationship with metabolism. Molecular and cellular biology 30: 5636–5648. 20937769 10.1128/MCB.00781-10 PMC3004277

[51] Rey G, Cesbron F, Rougemont J, Reinke H, Brunner M, et al (2011) Genome-wide and phase-specific DNA-binding rhythms of BMAL1 control circadian output functions in mouse liver. PLoS biology 9: e1000595. 21364973 10.1371/journal.pbio.1000595 PMC3043000

[52] Finck BN, Kelly DP (2006) PGC-1 coactivators: inducible regulators of energy metabolism in health and disease. J Clin Invest 116: 615–622. 16511594 10.1172/JCI27794 PMC1386111

[53] Lin J, Handschin C, Spiegelman BM (2005) Metabolic control through the PGC-1 family of transcription coactivators. Cell Metab 1: 361–370. 16054085 10.1016/j.cmet.2005.05.004

[54] Rhee J, Inoue Y, Yoon JC, Puigserver P, Fan M, et al (2003) Regulation of hepatic fasting response by PPARgamma coactivator-1alpha (PGC-1): requirement for hepatocyte nuclear factor 4alpha in gluconeogenesis. Proceedings of the National Academy of Sciences of the United States of America 100: 4012–4017. 12651943 10.1073/pnas.0730870100 PMC153039

[55] Yoon JC, Puigserver P, Chen G, Donovan J, Wu Z, et al (2001) Control of hepatic gluconeogenesis through the transcriptional coactivator PGC-1. Nature 413: 131–138. 11557972 10.1038/35093050

[56] Reddy JK, Hashimoto T (2001) Peroxisomal beta-oxidation and peroxisome proliferator-activated receptor alpha: an adaptive metabolic system. Annual review of nutrition 21: 193–230. 10.1146/annurev.nutr.21.1.193 11375435

[57] Ma C, Agrawal G, Subramani S (2011) Peroxisome assembly: matrix and membrane protein biogenesis. The Journal of cell biology 193: 7–16. 21464226 10.1083/jcb.201010022 PMC3082194

[58] Platta HW, Erdmann R (2007) Peroxisomal dynamics. Trends in cell biology 17: 474–484. 17913497 10.1016/j.tcb.2007.06.009

[59] Platta HW, Girzalsky W, Erdmann R (2004) Ubiquitination of the peroxisomal import receptor Pex5p. The Biochemical journal 384: 37–45. 15283676 10.1042/BJ20040572 PMC1134086

[60] Kiel JA, Emmrich K, Meyer HE, Kunau WH (2005) Ubiquitination of the peroxisomal targeting signal type 1 receptor, Pex5p, suggests the presence of a quality control mechanism during peroxisomal matrix protein import. The Journal of biological chemistry 280: 1921–1930. 15536088 10.1074/jbc.M403632200

[61] Kragt A, Voorn-Brouwer T, van den Berg M, Distel B (2005) The Saccharomyces cerevisiae peroxisomal import receptor Pex5p is monoubiquitinated in wild type cells. The Journal of biological chemistry 280: 7867–7874. 15632140 10.1074/jbc.M413553200

[62] Rucktaschel R, Girzalsky W, Erdmann R (2011) Protein import machineries of peroxisomes. Biochimica et biophysica acta 1808: 892–900. 20659419 10.1016/j.bbamem.2010.07.020

[63] Li S, Liu C, Li N, Hao T, Han T, et al (2008) Genome-wide coactivation analysis of PGC-1alpha identifies BAF60a as a regulator of hepatic lipid metabolism. Cell metabolism 8: 105–117. 18680712 10.1016/j.cmet.2008.06.013 PMC2578827

[64] Kulozik P, Jones A, Mattijssen F, Rose AJ, Reimann A, et al (2011) Hepatic deficiency in transcriptional cofactor TBL1 promotes liver steatosis and hypertriglyceridemia. Cell metabolism 13: 389–400. 21459324 10.1016/j.cmet.2011.02.011

[65] Purushotham A, Schug TT, Xu Q, Surapureddi S, Guo X, et al (2009) Hepatocyte-specific deletion of SIRT1 alters fatty acid metabolism and results in hepatic steatosis and inflammation. Cell metabolism 9: 327–338. 19356714 10.1016/j.cmet.2009.02.006 PMC2668535

[66] Finck BN, Gropler MC, Chen Z, Leone TC, Croce MA, et al (2006) Lipin 1 is an inducible amplifier of the hepatic PGC-1alpha/PPARalpha regulatory pathway. Cell metabolism 4: 199–210. 16950137 10.1016/j.cmet.2006.08.005

[67] Mitsui S, Yamaguchi S, Matsuo T, Ishida Y, Okamura H (2001) Antagonistic role of E4BP4 and PAR proteins in the circadian oscillatory mechanism. Genes & development 15: 995–1006. 11316793 10.1101/gad.873501 PMC312673

